# Integrated effects of dietary supplementation of *Spirulina platensis* in working dogs: nutritional, biochemical, antioxidant, immunological, gut-related microbial and nutrigenomic insights

**DOI:** 10.1186/s12917-026-05498-5

**Published:** 2026-05-06

**Authors:** Hala A. Abdelhady, Aya M. Yassin, Hassan Aboul-Ella, Khaled Nasr El-din Fahmy, Haithem A. M. Farghali, Tony M.A.

**Affiliations:** 1 Department of Nutrition and Clinical Nutrition, Faculty of Veterinary Medicine, Cairo University, Giza, Egypt; 2 Department of Biochemistry and Molecular Biology, Faculty of Veterinary Medicine, Cairo University, Giza, Egypt; 3 Department of Microbiology, Faculty of Veterinary Medicine, Cairo University, Giza, Egypt; 4 Department of Surgery, Anesthesiology and Radiology, Faculty of Veterinary Medicine, Cairo University, Giza, Egypt

**Keywords:** Antioxidant, Calprotectin, Dogs, FABP2, Fecal IgA, Fecal microbiota, Intestinal health, Occludin, *Spirulina platensis*, TRAF6

## Abstract

**Background:**

The present study investigated the effects of daily dietary supplementation with *Spirulina platensis* powder at two inclusion levels (0.04 g and 0.08 g/kg body weight) for working dogs. *Spirulina platensis* is a nutrient-rich microalgae containing high-quality protein, vitamins, minerals, and antioxidant pigments. These constituents are reported to possess immunomodulatory, antioxidant, and gut health-promoting effects. Dose-dependent effects on nutritional status, metabolic profiles, antioxidant and inflammatory status, gut integrity, immune function, and systemic gut health-related nutrigenomic responses were assessed in working dogs. A total of 15 adult male German Shepherd working dogs (age 2–3 years, weight range 24–26.5 kg) were randomly assigned to 3 groups (n = 5) over a 7-week experimental period, with evaluations at weeks 0, 3, and 7. The groups were as follows: first group, control (CON), fed on a basal diet; the second group (SP1) fed on the basal diet plus 0.04 g/kg body weight/day *Spirulina* powder; the third group (SP2) fed on the basal diet plus 0.08 g/kg body weight /day *Spirulina* powder.

**Results:**

Body weight, body condition score, daily food intake, fecal score, and fecal moisture did not differ among the three groups during the trial (*P* > 0.05). Indeed, *Spirulina* supplementation was significantly associated with higher serum total protein, albumin, and globulin concentrations (*P* < 0.05). Moreover, a significant difference in lipid profile was observed, characterized by reduced total cholesterol, triacylglycerol, low-density lipoprotein (LDL), and very low-density lipoprotein (VLDL)*,* and increased high-density lipoprotein (HDL) levels (*P* < 0.05). Similarly, lower aspartate aminotransferase (AST), alanine aminotransferase (ALT), and urea concentrations were observed in *Spirulina-*supplemented groups (*P* < 0.05)*.* Furthermore, supplemented groups exhibited enhanced total antioxidant capacity (T-AOC) and reduced malondialdehyde (MDA) concentrations (*P* < 0.05). Regarding serum gut barrier integrity-related genes, there is a significant upregulation of occludin (OCLN) and a reduction in fatty acid binding protein 2 (FABP2) in the supplemented groups (*P* < 0.05)*.* In terms of inflammatory and immune biomarkers, there is significant downregulation of interleukin-6 (IL-6) and tumor necrosis factor-receptor associated factor 6 (TRAF6) in the serum of dogs supplemented with *Spirulina* (*P* < 0.05)*. *Interestingly, *Spirulina-*supplemented groups showed a significant decrease in calprotectin and increase in fecal IgA levels (*P* < 0.05). In addition, Changes in selected culturable fecal bacterial populations were observed in the *Spirulina-s*upplemented groups indicated by significant increased total bacterial count and *lactobacillus* count accompanied by a lower coliform level (*P* < 0.05). For the parameters that showed significant differences, treatment, time, treatment × time interaction were all significant (*P* < 0.05). In most parameters, the higher inclusion level (0.08 g/kg) produced more pronounced effects.

**Conclusion:**

Short-term dietary supplementation of *Spirulina platensis*, particularly at 0.08 g/kg body weight inclusion level, was associated with modulation of biochemical profile, antioxidant status, inflammatory and immune biomarkers, selected microbial markers, and overall intestinal health in working dogs. These findings suggest potential systemic biological effects; however, considering the limited sample size and exploratory design, the results should be interpreted cautiously and warrant confirmation in larger, longer-term studies.

**Graphical Abstract:**

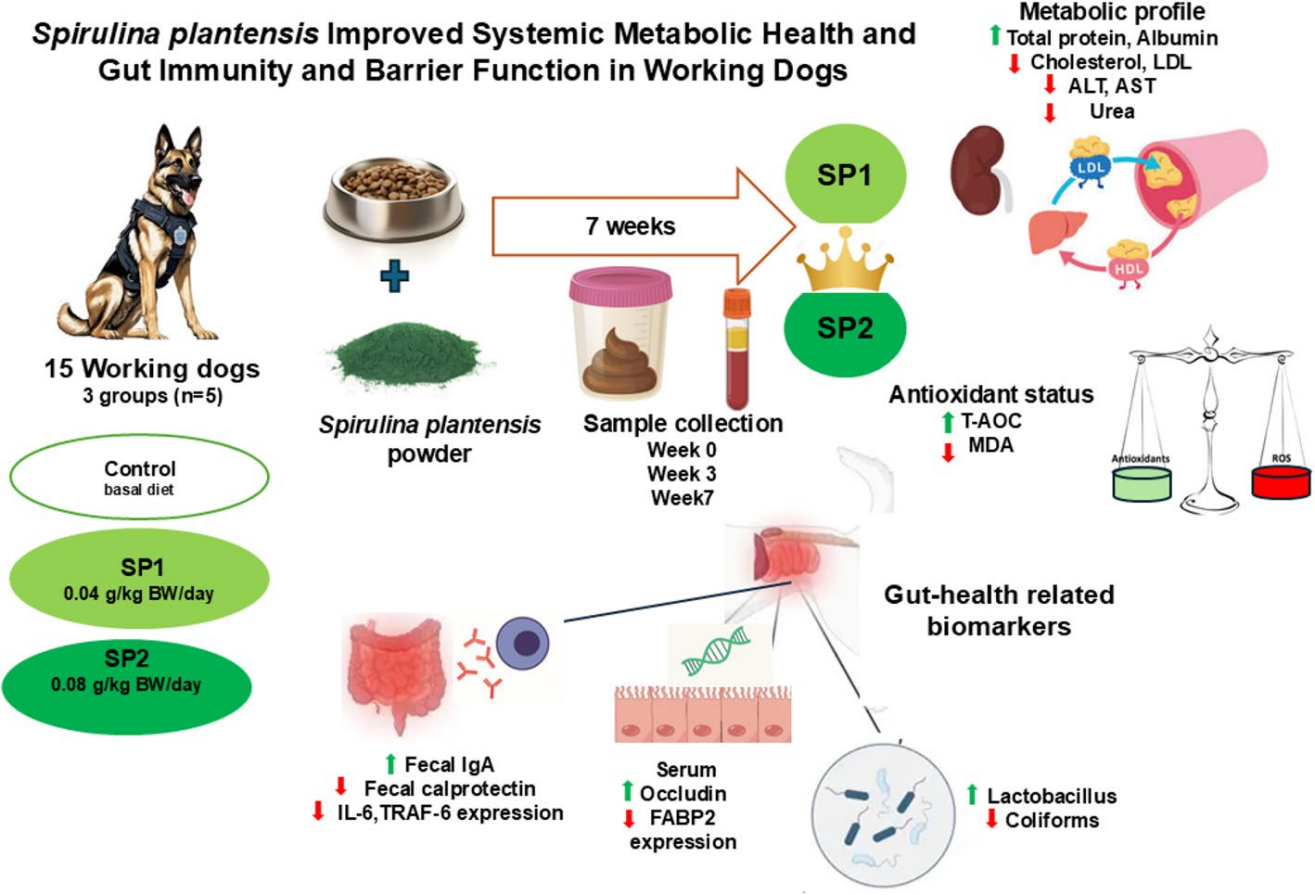

## Introduction

Working dogs, such as those used in search and rescue, law enforcement, military, and competitive sports, face significant physical and mental strain. These animals frequently experience stressful events, environmental stressors (heat, cold, and terrain variability), psychological stress related to task performance and training, and altered feeding and rest patterns, all of which differ substantially from the lifestyle of companion (pet) dogs. Previous studies have demonstrated that these cumulative stressors are associated with sustained activation of stress-responsive neuroendocrine pathways. Such conditions have been shown to influence the oxidative stress markers, modulation of immune parameters, and alterations in gastrointestinal function and gut microbiota composition [1–3]. Therefore, working dogs represent a biologically relevant and sensitive model for evaluating the effects of dietary interventions aimed at improving antioxidant defense, immune competence, and gut health, particularly under conditions of elevated physiological stress. Nutritional approaches that can boost immunological function, increase physiological resilience, enhance performance, and recovery outcomes in these animals are therefore gaining attention. In this context, the use of nutraceuticals has attracted growing interest in canine health management, referring to nutrient-based foods or supplements that exert health-promoting effects beyond their fundamental nutritional roles [4, 5]. Among these, the blue-green microalga *Spirulina* (*Spirulina plantensis*) has emerged as a promising functional ingredient due to its high content of bioactive chemicals. *Spirulina platensis* is a multicellular, filamentous, and photosynthetic cyanobacterium with a spiral shape. that has been marketed and consumed as a food for humans and animals [6]. *Spirulina* possesses an exceptional nutritional profile, containing 60–70% high biological value crude protein. It is also rich in vitamins, minerals, essential fatty acids, and antioxidant pigments [7]. These bioactive compounds are linked with broad-spectrum and numerous health benefits, including immunomodulatory, hypolipidemic, antioxidant, anti-inflammatory, hepatoprotective, nephroprotective, anti-viral, and anti-bacterial effects [7–11]. Owing to these properties, *Spirulina* has been increasingly incorporated as a nutraceutical feed additive in animal nutrition, with growing interest in its potential to modulate metabolic, immunological, and gut-related molecular pathways under physiologically demanding conditions [12–14].

Nutrigenomics is an interdisciplinary field that integrates nutritional science with molecular biology to investigate how specific nutrients modulate gene expression and physiological responses within a given population [15]. Understanding the nutrigenomic effects of nutraceuticals can reveal the mechanisms of their physiological and health-promoting actions, particularly in relation to gut integrity, immune regulation, and inflammatory pathways.

Emerging evidence in canine nutritional physiology indicates that dietary bioactive compounds can influence molecular pathways involved in inflammatory regulation, oxidative balance, and intestinal barrier function, particularly under conditions of increased physiological demand [16–18]. Working dogs exposed to sustained stress may exhibit alterations in inflammatory signaling mediators and epithelial integrity markers, which are increasingly recognized as nutrigenomically responsive targets [19].

There are several studies on the incorporation of *Spirulina plantensis* as a nutraceutical in the diets of both humans and animals for its remarkable effects on general health, immune status, antioxidant activity, lipid profile, and gut immune health [11, 12, 20–24]. Previous studies have evaluated *Spirulina* supplementation in dogs across a range of doses (0.04–0.19 g/kg BW/day) and these inclusion levels were reported to be well tolerated and palatable, with no significant adverse effects on gastrointestinal parameters, behavior, or general health status [7]. More recently, Stefanutti et al. [11] showed that *Spirulina* supplementation in overweight dogs over 12 weeks enhanced antioxidant status and exerted beneficial metabolic effects, including reductions in triglycerides and bilirubin concentrations. Importantly, no negative effects on health or weight management were observed.

Although *Spirulina* has been widely investigated for its health-promoting effects in various animal species [12, 25, 26], to date, limited in vivo studies have evaluated the effects of dietary supplementation of *Spirulina platensis* powder in working dogs. In particular, there is a lack of integrated studies evaluating gut-related nutrigenomic responses, fecal microbiota composition, metabolic as well as inflammatory and immunological biomarkers, and physiological performance. Moreover, dose-dependent responses of *Spirulina platensis* supplementation in working dogs have not been sufficiently characterized.

Based on this concept, we hypothesized that dietary supplementation of *Spirulina platensis* would improve the nutritional, biochemical, antioxidant, and immunological parameters in adult working dogs in a dose-dependent manner.

Accordingly, the present study aimed to evaluate and compare the effects of dietary supplementation of *Spirulina platensis* powder at two inclusion levels for adult working dogs. The objectives of the present study were to assess the potential dose-dependent responses in selected nutritional status parameters and fecal characteristics, protein profile and lipid metabolism, liver and kidney function, antioxidant biomarkers and fecal microbiota composition of adult working dogs. In addition, the study investigated some selected genes related to gut integrity (OCLN, FABP2) and systemic inflammatory and immunological (IL-6, TRAF6) response to determine the potential nutrigenomic impact of *Spirulina platensis* in dogs.

## Materials and Methods

### Ethical approval

The study was approved by the Institutional Animal Care and Use Committee (IACUC), Faculty of Veterinary Medicine, Cairo University. Approval No. is (VET CU110520251096). All experimental and animal management procedures in this study followed the ARRIVE guidelines.

### Animals and Experimental design

This study was designed as a randomized controlled trial with repeated measurements over time. A total of 15 healthy adult male German Shepherd dogs (age 2–3 years, weight range 24–26.5 kg) were randomly assigned to 3 groups (5 dogs/group). Dogs were randomly allocated to treatment groups using a random draw method. Allocation was performed by an investigator not involved in data collection or analysis to minimize selection bias and ensure baseline homogeneity among groups. Group assignments were concealed until the beginning of the supplementation period. The groups were as follows: first group, control (CON), fed on a balanced commercial diet. Second group (SP1) fed on the same balanced commercial diet mixed evenly with *Spirulina* powder with an inclusion level of 0.04 g/kg body weight/day. Third group (SP2) fed on the same balanced commercial diet mixed evenly with *Spirulina* powder with an inclusion level of 0.08 g/kg body weight/day.

### Rationale for inclusion levels selection

*Spirulina* (*Spirulina platensis*) was administered to the experimental groups at two levels: 0.04 g/kg body weight/day (SP1) and 0.08 g/kg body weight/day (SP2). The lower inclusion level (0.04 g/kg body weight/day) was selected to align with commonly reported effective supplementation levels in canine studies, while the higher inclusion level (0.08 g/kg body weight/day) was chosen to evaluate potential dose-dependent effects. These inclusion levels are supported by prior research in dogs, which reported safe and effective supplementation ranging from (0.06–0.08 g/kg/day) [7, 11]. In addition, human studies showed that daily intake of up to 10 g for six months (~ 0.14 g/kg/day for a 70 kg adult) is well tolerated without adverse effects [27]. The Food and Drug Administration (FDA) has also included *Spirulina* in the GRAS (Generally recognized as safe) list and recommends daily consumption of 0.1–6 g through various food products [28]. *Spirulina* was administered daily by incorporation into regular meals for the duration of the study, allowing evaluation of both standard and elevated supplementation levels while ensuring safety and scientific rigor**.**

The experimental study was conducted in a kennel for working dogs {Pro Doggy (K9 unit), Cairo, Egypt}**.** The experimental period lasted 7 weeks, preceded by a 1-week adaptation phase. Before the start of the trial, all dogs used had physical and clinical evaluations to determine their fitness for the research.

The dogs enrolled in this study were classified as working dogs and were engaged in odor-based detection, including narcotics and explosives detection work. Throughout the trial, all dogs follow a structured daily routine that ensures their physical well-being, mental stimulation, and social development. Each aspect of their day is carefully managed to support their training and readiness for working roles. The same veterinarian examined the dogs every day along the duration of the trial.

### Diet and housing

During the adaptation period, all dogs were fed on a commercial, nutritionally complete and balanced extruded dry dog food (Jessie®, Belgium) which met the nutritional requirements recommended by NRC [29]. The *Spirulina* used in this study is *Spirulina plantensis* in powder form. It was obtained from Algal Biotechnology unit, National Research Center, Dokki, Giza, Egypt. *Spirulina* powder was evenly sprinkled and attached to the top of the diet immediately before offering the meal to each dog to ensure precise individual dosing and voluntary complete consumption of *Spirulina* powder [30].

The *Spirulina platensis* powder used in this study was produced according to the standardized procedures to preserve its bioactive compounds. Briefly, the harvested biomass was washed, concentrated, and then gently dried at a controlled temperature of 40–45 °C to avoid degradation of heat-sensitive nutrients, such as phycocyanin, vitamins, and antioxidants [31, 32]. The dried biomass was then ground into a fine powder and stored at 4 °C in airtight containers until use.

The proximate composition of the basal diet and the used *Spirulina* was determined before the onset of the experiment in duplicate following the Association of Official Analytical Chemists methods [33]. Briefly, analyses included moisture, ash, ether extract, crude fiber and Kjeldahl nitrogen (Kjeldahl N), with crude protein (CP) calculated as Kjeldahl N × 6.25. Nitrogen-free extract (NFE) was calculated by difference on an as-fed basis as: 100 − (moisture % + crude protein % + crude fiber % + ether extract % + ash %) [29]. The chemical composition of the basal diet and the used *Spirulina* powder is illustrated in (Table 1) and (Table 2) respectively. All values were expressed on an as-fed basis, including moisture content.

**Table 1:** Chemical composition of experimental diets (As-fed basis):

Item	CON diet	SP1 diet	SP2 diet
Moisture%	9	9	9
CP%	28	28.07	28.13
EE%	14	13.98	13.96
CF%	3.5	3.49	3.49
Ash%	8.5	8.5	8.5
NFE%	37	36.96	36.92
ME (Kcal/100 g)	346.5	346.44	346.34

CON Control basal diet, SP1: basal diet plus *Spirulina platensis *powder 0.04 g/kg BW, SP2: basal diet plus *Spirulina platensis* powder 0.08 g/kg BW

*CP* Crude protein, *EE* Ether extract, *CF* Crude fiber, *NFE* Nitrogen free extract, *ME* Metabolizable energy

**Table 2:** Chemical composition of *Spirulina platensis* powder (As-fed basis)

Item	*Spirulina platensis* powder
Moisture%	8.5
CP%	60.5
EE%	3.2
CF%	0.1
Ash%	9
NFE%	18.7

CP Crude protein, EE Ether extract, CF Crude fiber, NFE Nitrogen free extract

The daily amount of food offered to each dog was calculated based on the calculated metabolizable energy (ME) requirements for adult working dogs with light-to-moderate physical activity as suggested by previously published data on odor-detection working dogs [34]. This was adjusted according to the ME density of the basal diet that was calculated using the modified Atwater factors based on as-fed basis [29, 35]:

$$\text{ME of food }\left(\mathrm{kcal}/\text{g food}\right)=\left\{\left(\mathrm{CP}{\%}\times 3.5\right)+\left(\mathrm{NFE}\%\times 3.5\right)+\left(\mathrm{EE}\%8.5\right)\right\}/100$$

Resting energy requirements (RER) = 70 × BW_kg_^0.75^

Daily energy requirements (DER) in Kcal = RER × 2.

The amount of food/dog/day (g/d) = DER (kcal/d) ÷ Diet ME (kcal/g).

Dogs were housed in individual cages (cage/dog code). Each dog in all different treatment groups was-fed individually once daily, and the amount was consumed within 30 min and adjusted weekly. Feeding once per day has been reported as safe for adult dogs [36]. Fresh clean drinking water was freely available during entire trial period.

### Nutritional status parameters and fecal characteristics

In weeks 0, 3 and 7, the following parameters were estimated by the same veterinarian: body weight (BW), body condition score (BCS), daily food intake, fecal score (FS), fecal moisture. BCS on a scale of one (too thin) to nine (too heavy) and a score of 4 or 5 represents the ideal score, according to the table of the WSAVA Global Nutrition Guidelines [37]. FS was assigned to fresh feces weekly by the same veterinarian using the Purina Fecal Scoring Chart ranging from one (very hard) to seven (watery) [38].

### Sample Collection and preparation

Sampling was performed 3 times at the beginning (Week 0; W0), middle (Week 3; W3) and at the end of the experiment (Week 7; W7). Blood samples were collected from the cephalic vein of each dog into plain vacutainer tubes. Centrifugation of the blood specimens was performed (3500 rpm for 15 min) for separation of serum. The separated serum was stored at −80°C until analysis. Fresh fecal samples were collected using a sterile spatula and stored in a sterile plastic cup (cage/dog code). The collected fecal samples were processed and preserved at −80 °C until analysis for microbiota analyses and for the determination of fecal secretory IgA(sIgA) and calprotectin using commercial ELISA kit. In addition, Fecal moisture was analyzed by following Association of Official Analytical Chemists protocols [33].

### Serum biochemical indices

The serum samples were examined spectrophotometrically using (UV-2100 spectrophotometer (USA) for investigation of the following:

### Protein profile

Total protein and albumin concentration were determined using commercial kits and according to the manufacturer's instructions (Bio Diagnostic, Giza, Egypt) at wavelengths 550 nm and 630 nm, respectively. Globulin concentration was calculated as total protein minus albumin and A/G ratio according to [39].

### Lipid profile

The lipid profile is estimated using commercial kits provided by Bio diagnostic, Egypt. Total cholesterol (TC), TAG (Triacylglycerol), and high-density lipoprotein (HDL) cholesterol concentrations, were estimated following the methods described by [40–42]; respectively, while low-density lipoprotein (LDL), and very low-density lipoprotein (VLDL) cholesterol concentrations were calculated according to [43].

VLDL cholesterol concentration = Triglycerides/5.

LDL cholesterol concentration = Total cholesterol – (HDL + VLDL).

### Liver and kidney function tests

Liver function parameters (ALT, and AST) were evaluated in serum using commercial kits (Spectrum, Egypt) at 340 nm, based on the method established by [40]. Kidney function tests, including urea and creatinine levels, were measured according to the procedures described by [44, 45]; respectively, using commercial kits (Bio Diagnostic, Giza, Egypt).

### Redox status

Serum malondialdehyde (MDA) concentration was used as an index of lipid peroxidation as described by [46]. MDA was determined by measuring the thiobarbituric acid reactive species. The absorbance was measured at 534 nm. The concentration of serum total antioxidant capacity (T-AOC) was measured using commercial assay kits (Bio Diagnostic, Giza, Egypt), following the protocol described by [47] at 505 nm.

### Quantitative real-time PCR analysis of Gut health-related nutrigenomic response

Gene expression levels of some gut health-related nutrigenomic genes including OCLN, FABP2, IL-6, TRAF6 were quantified by quantitative real-time PCR (qRT-PCR). Total RNA was extracted from the serum samples, using a total RNA purification kit (Jena Bioscience, Germany, Cat. #PP-210S). RNA concentration and purity were verified using the Nanodrop ND-1000 Spectrophotometer (Thermo Scientific, USA). Reverse transcription and cDNA synthesis were performed by the Revert Aid First Strand cDNA Synthesis Kit (Thermo Scientific, USA, Cat. #K1622).

The relative mRNA expression levels were determined using iQ SYBR® Green Supermix (Bio-Rad 1,708,880, USA). Primers of the tested genes are listed in (Table 3), and β-actin (ACTB) was used as an endogenous reference gene. The PCR cycling conditions were as follows: a three-minute denaturation phase at 95 °C followed by 40 cycles that included denaturation (95°C for 15 s), annealing (60°C for 30 s), and extension (72°C for 30 s). Following amplification, melting curve analysis was used to confirm PCR product specificity. Each experiment consisted of three technical replicates and a no-template control (NTC). Relative gene expression was determined using the 2 − ΔΔCT technique [48].

**Table 3:** Primers for Real-time PCR for tested genes

**Genes**	**Forward primer (**5'**- > **3'**)**	**Reverse primer (**5'**- > **3'**)**	**Product size**	**REF./accession No**
GAPDH	AGGTCGGAGTCAACGGATTT	ATCTCGCTCCTGGAAGATGG	230	[49]
OCLN	TCTGACTAGGAGTTCGGGGA	CCCAGGAAGGCACTCAGTATTATT	261	NM_001003195.1
FABP2	TGGAGCATTGACTTGAGGTGAG	TGCTTGATTCCTTGACGGTGA	225	XM_038444525.1
IL6	ACCACTCACCTCTGCAAACA	GCTGAAACTCCACAAGACCG	236	NM_001003301.1
TRAF6	GTCATTCACAGCCCTGGATTCT	AGCTCTGGTTTGGCATCCATTA	248	XM_003432322.5

*GAPDH* glyceraldehyde−3-phosphate dehydrogenase, *OCLN* Occludin, *FABP2* fatty acid binding protein 2, *IL-6* interleukin-6, *TRAF6* TNF receptor-related factor 6

### Fecal secretory IgA(sIgA) and calprotectin

Preparation of fecal samples was done according to the manufacturer’s protocol. Briefly, approximately 1 g of feces was rinsed three times with Phosphate-buffered saline (PBS) (w:v = 1:9), sonicated, then centrifuged at 5000 × g for 10 min, after which the supernatant was collected and stored at −80 °C till analysis [1]. Fecal secretory IgA concentrations were quantified by commercial ELISA kit (Dog IgA ELISA kit, Cat No ELK0977, ELK Biotechnology, Wuhan, China). Fecal calprotectin levels were measured using ELISA kit (Dog CALP ELISA kit, Cat No. ELK824, ELK Biotechnology, Wuhan, China). Optical density was measured at 450 nm, and the calprotectin and fecal IgA concentrations were calculated in accordance with the manufacturer’s instructions.

### Fecal microbiota analysis

The total bacterial counts from fecal samples were performed as follows: One gram of the fecal sample was serially diluted (ten-fold dilution) using sterile saline. 100µl of each dilution was plated on plate count agar (PCA, Himedia®, India for viable counts. Following inoculation, the plates were incubated for 24 h at 37 °C in aerobic conditions. Colonies were counted and reported as the logarithm of colony-forming units per gram (log10 CFU/g) following incubation [50].

The method used to quantify the *lactobacillus* and coliform bacteria was performed as follows: One gram of the fecal sample was serially diluted (ten-fold dilution) using sterile saline. 100µl of each dilution was plated on MacConkey agar (Himedia®, India) plates for coliform bacteria counts and De Man, Rogosa, and Sharpe (MRS) agar (Himedia®, India) plates for *Lactobacillus* spp. counts to obtain a viable count. Following inoculation, the MRS and MacConkey agar plates were incubated for 24–48 h at 37 °C in anaerobic and aerobic conditions, respectively. Colonies of the coliform and *lactobacillus* bacteria were counted and reported as the logarithm of colony-forming units per gram (log10 CFU/g) following incubation [12, 51].

### Statistical Analysis

All data were analyzed using repeated-measures analysis of variance (GLM Repeated Measures) in SPSS version 20 software. Time (weeks 0, 3, and 7) was specified as the within-subject factor, and treatment group (CON, SP1, SP2) was specified as the between-subject factor. The model included the main effects of treatment and time, as well as the treatment × time interaction. The model used was as follows

$$\mathrm{Yijk}=\upmu +\mathrm{Ti}+\mathrm{Pj}+\left(\mathrm{T}\times \mathrm{P}\right)\mathrm{ij}+\mathrm{Ak}+\mathrm{eijk}$$

where:

Yijk = observed value of the dependent variable, μ = overall mean, Ti = fixed effect of treatment, Pj = fixed effect of period/time, (T × P) ij = interaction between treatment and time, Ak = random effect of animal (subject), and eijk = residual error.

Sphericity was assessed using Mauchly’s test. When the sphericity assumption was violated, Greenhouse–Geisser corrections were applied and corrected P-values are reported. Normality of residuals was evaluated using Shapiro–Wilk tests and inspection of Q–Q plots. Homogeneity of variance between groups was assessed using Levene’s test.

When significant main effects or interactions were detected, pairwise comparisons were performed using Bonferroni-adjusted post hoc tests. Baseline (Week 0) equivalence among groups was assessed prior to longitudinal analysis. Data are presented as mean, SEM represents pooled standard error from the repeated-measures model, and differences were considered statistically significant at *P* < 0.05.

Although the study included a relatively small number of dogs (n = 5 per group), similar sample sizes have been successfully used in previous canine studies investigating behavioral [52] and pharmacological [53] endpoints. Repeated measurements across multiple time points were performed to increase sensitivity and reduce inter-individual variability, allowing meaningful biological trends to be detected despite the limited sample size.

## Results

### Nutritional status parameters and fecal characteristics

All dogs remained healthy during the study, and no side effects (e.g., vomiting/diarrhea) were recorded. The results of body weight, body condition score, food intake, fecal score, and fecal moisture are presented in (Table 4).

**Table 4:** Effects of *Spirulina platensis* supplementation on nutritional status parameters and fecal characteristics at weeks 0, 3 and 7

Parameters	CON	SP1	SP2	SEM	*P*-value			Partial η^2^ (Interaction)
					**Treatment**	**Time**	**Treatment × Time**	
Body weight (kg)								
Week 0	25	25.3	25	0.37	0.910	< 0.001	0.233	0.21
Week 3	25.3	25.4	25.4	0.36				
Week 7	25.5	25.8	25.8	0.36				
BCS (1–9 scale)								
Week 0	4	4.01	4.02	0.04	0.258	0.026	0.288	0.18
Week 3	4.1	4.1	4.2	0.1				
Week 7	4.1	4.1	4.4	0.1				
Daily food intake (g/d)								
Week 0	451.68	455.72	451.68	5.04	0.91	< 0.001	0.23	0.22
Week 3	455.96	457.08	457.12	4.94				
Week 7	458.74	462.46	462.76	4.97				
Fecal score (1–5 scale)								
Week 0	2.7	2.8	2.8	0.12	0.931	0.369	0.988	0.01
Week 3	2.7	2.6	2.6	0.11				
Week 7	2.8	2.8	2.9	0.12				
Fecal moisture %								
Week 0	74.52	71.06	69.73	1.69	0.130	< 0.001	0.421	0.13
Week 3	71.5	68.08	66.98	1.79				
Week 7	64.41	63.97	62.645	0.6				

CON Control: basal diet, SP1: basal diet plus *Spirulina platensis* powder 0.04 g/kg BW, SP2: basal diet plus *Spirulina platensis* powder 0.08 g/kg BW

Data represented as mean value

SEM represents pooled standard error of mean from the repeated-measures model where (*n* = 5)

Values in the same row with different lowercase superscripts (a, b, c) are significantly different at *P* < 0.05

*BCS* body condition score

Mauchly’s test indicated violation of the sphericity assumption for time in the Body weight, daily food intake, and fecal moisture % parameters (*P* < 0.001); therefore, Greenhouse–Geisser corrected results are reported.

Body weight increased significantly over time (*P* < 0.001); however, no significant treatment effect (*P* = 0.910) or treatment × time interaction (*P* = 0.233) was observed. Similarly, daily food intake showed a significant time effect (*P* < 0.001) but was not influenced by treatment (*P* = 0.910) or the interaction term (*P* = 0.230).

Body condition score (BCS) showed moderate time effect (*P* = 0.026), while neither the treatment (*P* = 0.258) nor time × treatment interaction (*P* = 0.288) showed statistical significance. Fecal score showed stability throughout the study, with no significant effects of time (*P* = 0.369), treatment (*P* = 0.931), or their interaction (*P* = 0.988). Meanwhile, the fecal moisture % decreased significantly over time (*P* < 0.001), but no significant treatment (*P* = 0.130) or treatment × time interaction (*P* = 0.421) effects were detected.

### Serum biochemical indices

The results of all groups were within the normal laboratory range for healthy dogs. At the beginning of the experiment (Week 0), no significant differences were observed among the groups for any of the serum parameters analyzed.

### Protein profile

Serum total protein concentrations were significantly influenced by time, treatment, and their interaction (*P* < 0.001). *Spirulina* supplementation increased total protein levels compared with the control group, with more pronounced effects at the higher inclusion level (SP2). In addition, SP1 and SP2 showed progressive increases at Weeks 3 and 7.

For albumin, Mauchly’s test indicated violation of the sphericity assumption for time (*P* = 0.024); therefore, Greenhouse–Geisser corrected results are reported. Albumin concentrations were significantly affected by time, treatment, and treatment × time interaction (*P* < 0.001), with supplemented groups exhibiting higher levels compared with control, particularly at Week 7.

Regarding the globulin level, it was significantly affected by time (*P* = 0.001), treatment (*P* = 0.003), and their interaction (*P* = 0.026), Supplemented groups demonstrated higher globulin concentrations over time compared with the control. Meanwhile, the albumin-to-globulin (A/G) ratio was not significantly affected by time (*P* = 0.549), treatment (*P* = 0.363), or their interaction (*P* = 0.616**)** (Table 5).

**Table 5:** Effects of *Spirulina platensis* supplementation on protein profile at weeks 0, 3 and 7

Parameters	CON	SP1	SP2	*SEM*	*P*-value			Partial η^2^ (Interaction)
					**Treatment**	**Time**	**Treatment × Time**	
Total protein(g/dL)								
Week 0	6.9^a^	6.8^a^	6.85^a^	0.09	< 0.001	< 0.001	< 0.001	0.64
Week 3	6.79^b^	7.49^a^	7.83^a^	0.12				
Week 7	6.92^c^	7.59^b^	7.9^a^	0.07				
Albumin (g/dL)								
Week 0	3.28^a^	3.28^a^	3.28^a^	0.02	< 0.001	< 0.001	< 0.001	0.86
Week 3	3.28^c^	3.48^b^	3.75^a^	0.03				
Week 7	3.31^c^	3.51^b^	3.78^a^	0.02				
Globulin (g/dL)								
Week 0	3.61^a^	3.54^a^	3.56^a^	0.11	0.003	0.001	0.026	0.36
Week 3	3.5^b^	4^b^	4.08^a^	0.13				
Week 7	3.6^c^	4.08^b^	4.17^a^	0.08				
A/G ratio								
Week 0	0.91	0.93	0.93	0.03	0.363	0.549	0.616	0.10
Week 3	0.94	0.87	0.92	0.04				
Week 7	0.92	0.86	0.91	0.02				

CON Control: basal diet, SP1: basal diet plus *Spirulina platensis* powder 0.04 g/kg BW, SP2: basal diet plus *Spirulina platensis* powder 0.08 g/kg BW

Data represented as mean value

SEM represents pooled standard error of mean from the repeated-measures model where (*n* = 5)

Values in the same row with different lowercase superscripts (a, b, c) are significantly different at *P* < 0.05

### Lipid profile

Serum lipid parameters were significantly affected by time, group, and treatment × time interaction (*P* < 0.001 for all variables). Mauchly’s test indicated violation of the sphericity assumption for time in the total cholesterol (*P* = 0.007), HDL (*P* = 0.04), and LDL (*P* = 0.003); therefore, Greenhouse–Geisser corrected results are reported. Interestingly, Total cholesterol, Triacylglycerol, LDL, and VLDL showed a clear decreasing trend among the groups, with the SP2 group showing the lowest values and the CON group showing the highest values. HDL was significantly highest in the SP2 group, followed by the SP1 group, and the lowest values were observed in the CON group, particularly at Week 7 (Table 6).

**Table 6:** Effects of *Spirulina platensis* supplementation on lipid profile at weeks 0, 3 and 7

Parameters	CON	SP1	SP2	*SEM*	*P*-value			Partial η^2^ (Interaction)
					**Treatment**	**Time**	**Treatment × Time**	
Total cholesterol (mg/dL)								
Week 0	253^a^	253^a^	253^a^	1.24	< 0.001	< 0.001	< 0.001	0.91
Week 3	266^a^	243^b^	224^c^	1.64				
Week 7	270^a^	197^b^	161^c^	2.49				
TAG (mg/dL)								
Week 0	100.27^a^	100.25^a^	100.26^a^	1.66	< 0.001	< 0.001	< 0.001	0.9
Week 3	110.32^a^	90.86^b^	80.38^b^	1.16				
Week 7	115.23^a^	85.18^b^	70.47^c^	0.85				
HDL (mg/dL)								
Week 0	74.24^a^	76.13^a^	77.2^a^	0.69	< 0.001	< 0.001	< 0.001	0.89
Week 3	72.36^c^	84.28^b^	95.2^a^	0.71				
Week 7	70.16^c^	90.15^b^	100.57^a^	0.63				
LDL (mg/dL)								
Week 0	159.27^a^	157.37^a^	156.29^a^	1.3	< 0.001	< 0.001	< 0.001	0.85
Week 3	172.16^a^	140.56^b^	113.30^c^	1.72				
Week 7	177.19^a^	90.23^b^	46.6^c^	2.81				
VLDL (mg/dL)								
Week 0	20.05^a^	20.05^a^	20.05^a^	0.33	< 0.001	< 0.001	< 0.001	0.9
Week 3	22.06^a^	18.17^b^	16.07^b^	0.23				
Week 7	23.04^a^	17.05^b^	14.09^c^	0.17				

CON Control: basal diet, SP1: basal diet plus *Spirulina platensis*powder 0.04 g/kg BW, SP2: basal diet plus *Spirulina platensis *powder 0.08 g/kg BW

Data represented as mean value. SEM represents pooled standard error of mean from the repeated-measures model where (*n* = 5)

Values in the same row with different lowercase superscripts (a, b, c) are significantly different at *P* < 0.05

*TAG* Triacylglycerol, *HDL* high-density lipoprotein, *LDL* low density lipoprotein, *VLDL* very low-density lipoprotein

### Liver and kidney function tests

The activities of the liver enzymes differ significantly among the groups. Serum AST and ALT activities were significantly influenced by time, treatment, and treatment × time interaction (*P* < 0.001). *Spirulina*-supplemented groups exhibited significant reductions at Weeks 3 and 7, with the greatest decreases observed in SP2 compared with the CON group. Regarding kidney function, serum urea concentrations were significantly affected by time, treatment, and their interaction (*P* < 0.001). Urea levels remained relatively stable in the CON group but declined markedly in the *Spirulina*-supplemented groups, particularly in SP2 by Week 7.

In contrast, serum creatinine levels were not significantly influenced by time (*P* = 0.474), treatment (*P* = 0.831), or their interaction (*P* = 0.108) (Table 7).

**Table 7:** Effects of *Spirulina platensis* supplementation on liver and kidney function tests at weeks 0, 3 and 7

Parameters	CON	SP1	SP2	*SEM*	*P*-value			Partial η^2^ (Interaction)
					**Treatment**	**Time**	**Treatment × Time**	
Liver function tests								
AST (U/L)								
Week 0	41.98^a^	41.68^a^	42.12^a^	0.68	< 0.001	< 0.001	< 0.001	0.92
Week 3	45.39^a^	36^b^	30.61^c^	0.57				
Week 7	46.37^a^	33.1^b^	27.63^c^	0.45				
ALT(U/L)								
Week 0	34.94^a^	34.74^a^	35.1^a^	0.56	< 0.001	< 0.001	< 0.001	0.83
Week 3	34.92^a^	30^ab^	27.82^b^	0.48				
Week 7	35.67^a^	27.6^b^	25.2^c^	0.37				
Kidney function tests								
Urea (mg/dL)								
Week 0	42.16^a^	41.77^a^	41.72^a^	0.49	< 0.001	< 0.001	< 0.001	0.81
Week 3	43.48^a^	34.55^b^	32.15^c^	0.45				
Week 7	43.99^a^	25.5^b^	24.31^b^	0.31				
Creatinine (mg/dL)								
Week 0	1.29	1.26	1.27	0.02	0.831	0.474	0.108	0.06
Week 3	1.28	1.26	1.29	0.02				
Week 7	1.29	1.23	1.25	0.02				

CON Control: basal diet, SP1: basal diet plus *Spirulina platensis* powder 0.04 g/kg BW, SP2: basal diet plus *Spirulina platensis* powder 0.08 g/kg BW

Data represented as mean value

SEM represents pooled standard error of mean from the repeated-measures model where (*n *= 5)

Values in the same row with different lowercase superscripts (a, b, c) are significantly different at *P* < 0.05

*AST* aspartate aminotransferase, *ALT* alanine aminotransferase

### Redox status

Total antioxidant capacity (T-AOC) and MDA were significantly affected by time, treatment, and treatment × time interaction (*P* < 0.001). Throughout the study, SP2 group maintained significantly improved redox status followed by SP1 group then CON group at both the middle and end of the study (*P* < 0.05). This improved redox status is characterized by enhanced total antioxidant activity (T-AOC) and reduced malondialdehyde (MDA) concentrations, particularly in SP2 at Week 7. (Table 8).

**Table 8:** Effects of *Spirulina platensis* supplementation on redox status at weeks 0, 3 and 7

Parameters	CON	SP1	SP2	SEM	*P*-value			Partial η^2^ (Interaction)
					**Treatment**	**Time**	**Treatment × Time**	
T-AOC (mM/mL)								
Week 0	0.8^a^	0.79^a^	0.79^a^	0.01	< 0.001	< 0.001	< 0.001	0.89
Week 3	0.79^c^	1.03^b^	1.27^a^	0.03				
Week 7	0.77^c^	1.23^b^	1.37^a^	0.02				
MDA (nmol/mL)								
Week 0	15.94^a^	15.9^a^	16.14^a^	0.32	< 0.001	< 0.001	< 0.001	0.9
Week 3	16.65^a^	14.7^b^	13.33^c^	0.24				
Week 7	16.89^a^	11.1^b^	9.4^c^	0.17				

CON Control: basal diet, SP1: basal diet plus *Spirulina platensis *powder 0.04 g/kg BW, SP2: basal diet plus *Spirulina platensis* powder 0.08 g/kg BW

Data represented as mean value

SEM represents pooled standard error of mean from the repeated-measures model where (*n* = 5)

Values in the same row with different lowercase superscripts (a, b, c) are significantly different at *P* < 0.05

*T-AOC* total antioxidant capacity, *MDA* malondialdehyde

### Gut health-related nutrigenomic response

**Fig. 1:**
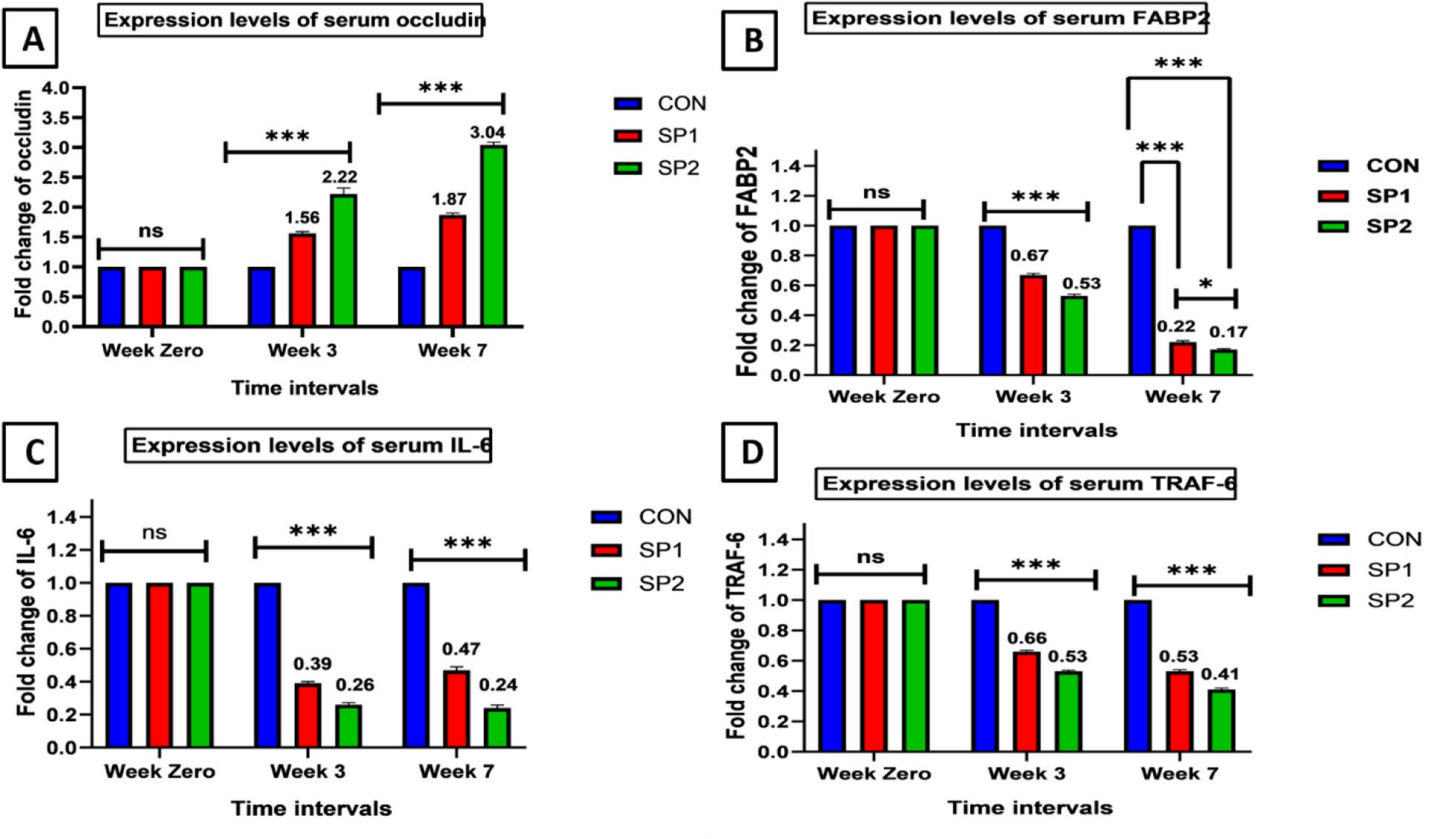
Effects of *Spirulina platensis* supplementation on gut health-related nutrigenomic response at weeks 0, 3 and. 7 (**A**) OCLN: occludin, **B** FABP2: fatty acid binding protein 2, **C** IL-6: interleukin −6, **D**TRAF 6: TNF receptor- associated factor 6. CON Control: basal diet, SP1: basal diet plus *Spirulina platensi*s powder 0.04 g/kg BW, SP2: basal diet plus *Spirulina platensis* powder 0.08 g/kg BW. Data represented as mean value ± standard error (S.E.) where (*n* = 5). Repeated-measures ANOVA revealed significant effects of time and treatment × time interaction (Greenhouse–Geisser corrected, *P* < 0.001). Significant differences were set by Bonferroni-adjusted post hoc test, where (ns) indicates non-significant; Asterisks (*) indicate the degree of statistical significance as follows (*): *P* < 0.05; (**): *P* < 0.01;(***): *P* < 0.001

The relative mRNA expression levels for genes that are related to the gut barrier function (OCLN and FABP2) and systemic inflammation (IL-6 and TRAF6) in serum are shown in (Fig. 1). At baseline (week 0), no significant differences were observed among the CON group and the *Spirulina* supplemented groups (SP1 and SP2) (*P* > 0.05) in all the measured gene expression biomarkers.

For OCLN, Mauchly’s test indicated violation of sphericity (*P* = 0.044); therefore, Greenhouse–Geisser–corrected results are reported. A significant effect of time, treatment and a treatment × time interaction was observed (*P* < 0.001, Partial η^2^ = 0.85). Post hoc analysis showed that OCLN expression increased significantly in *Spirulina*-supplemented groups, with the greatest upregulation observed in SP2 at Weeks 3 and 7 (Fig. 1 A)**.** Meanwhile, FABP2 showed a significant effect of time, treatment, and treatment × time interaction (*P* < 0.001, Partial η^2^ = 0.87). FABP2 expression was progressively reduced in *Spirulina*-supplemented groups, with the lowest levels observed in SP2. Differences among all groups were significant at week 7 (Fig. 1 B).

Regarding the inflammatory biomarker, IL-6 (Fig. 1 C) and TRAF6 (Fig. 1 D), both genes showed significant time, treatment effects and treatment × time interaction (*P* < 0.001, Partial η^2^ = 0.86 and 0.85, respectively). *Spirulina* supplementation was associated with significant downregulation of IL-6 and TRAF6, with more pronounced reductions in the high-inclusion level group at Weeks 3 and 7.

### Fecal sIgA and calprotectin

The effects of *Spirulina platensis* supplementation on markers of mucosal immunity and inflammation are summarized in (Table 9). Fecal secretory IgA (sIgA) concentrations were significantly influenced by time (*P* < 0.001), treatment (*P* = 0.03), and treatment × time interaction (*P* = 0.008). *Spirulina* supplementation resulted in marked increases at Weeks 3 and 7, with the highest levels observed in SP2.

**Table 9:** Effect of *Spirulina platensis* supplementation on fecal secretory IgA and fecal calprotectin at weeks 0, 3 and 7

Parameters	CON	SP1	SP2		SEM	*P*-value			Partial η^2^ (Interaction)
						**Treatment**	**Time**	**Treatment × Time**	
Fecal sIgA (ng/mL)									
Week 0	31.35^a^	31.25^a^	31.08^a^		0.17	0.03	< 0.001	0.008	0.52
Week 3	217.25^a^	244.97^a^	313.01^a^	38.10					
Week 7	177.86^b^	211.52^b^	360.39^a^	29.49					
Calprotectin (ng/mL)									
Week 0	52.13^a^	45.20^a^	48.68^a^	1.90		< 0.001	0.001	< 0.001	0.92
Week 3	85.0^a^	24.78^b^	19.64^b^	3.71					
Week 7	120.0^a^	44.42^b^	23.06^c^	8.06					

CON Control: basal diet, SP1: basal diet plus *Spirulina platensis* powder 0.04 g/kg BW, SP2: basal diet plus *Spirulina platensis* powder 0.08 g/kg BW

Data represented as mean value

SEM represents pooled standard error of mean from the repeated-measures model where (*n* = 5)

Values in the same row with different lowercase superscripts (a, b, c) are significantly different at *P* < 0.05

*SIgA* secretory IgA

Moreover**,** Calprotectin Mauchly’s test indicated violation of the sphericity assumption (*P* < 0.001); therefore, Greenhouse–Geisser corrected results are reported**.** Calprotectin concentrations were significantly affected by time (*P* = 0.001), treatment (*P* < 0.001), and their interaction (*P* < 0.001). In contrast to the control group, which showed progressive increases over time*, Spirulina*-supplemented groups exhibited substantial reductions, particularly in SP2 by Week 7.

### Fecal microbiota analysis

The analysis of fecal microbiota revealed a significant modulatory effect of *Spirulina* supplementation, as detailed in (Table 10). Mauchly’s test indicated violation of the sphericity assumption for time (*P* < 0.001); therefore, Greenhouse–Geisser corrected results are reported for total CFU. Total CFU was significantly affected by time, treatment, and treatment × time interaction (*P* < 0.001). *Spirulina* supplementation resulted in marked increases at Weeks 3 and 7, with the highest values observed in SP2. Both Coliform and *Lactobacillus* counts were also significantly influenced by time, treatment, and their interaction (P < 0.001). *Spirulina*-supplemented groups exhibited substantial reductions over time, particularly in SP2 for the coliform count. In contrast to *Lactobacillus,* which showed a pronounced increase at Weeks 3 and 7 compared with control, with the greatest elevation observed in the high-inclusion group SP2.

**Table 10:** Effects of *Spirulina platensis* supplementation on fecal microbiota at weeks 0, 3 and 7

Parameters log10 CFU/g	CON	SP1	SP2	SEM	*P*-value			Partial η^2^ (Interaction)
					**Treatment**	**Time**	**Treatment × Time**	
Total CFU								
Week 0	7.48^a^	7.55^a^	7.44^a^	0.15	< 0.001	< 0.001	< 0.001	0.91
Week 3	7.49^c^	8.33^b^	9.49^a^	0.08				
Week 7	7.57^c^	8.37^b^	9.55^a^	0.10				
Coliform								
Week 0	6.21^a^	6.21^a^	6.33^a^	0.24	< 0.001	< 0.001	< 0.001	0.81
Week 3	6.23^a^	5.34^b^	4.24^c^	0.01				
Week 7	6.24^a^	5.45^b^	4.27^c^	0.04				
*Lactobacillus*								
Week 0	4.25^a^	4.23^a^	4.24^a^	0.01	< 0.001	< 0.001	< 0.001	0.85
Week 3	4.29^c^	6.41^b^	7.42^a^	0.02				
Week 7	4.31^c^	6.45^b^	7.44^a^	0.02				

CON Control: basal diet, SP1: basal diet plus *Spirulina platensis* powder 0.04 g/kg BW, SP2: basal diet plus *Spirulina platensis* powder 0.08 g/kg BW

Data represented as mean value

SEM represents pooled standard error of mean from the repeated-measures model where (*n *= 5)

Values in the same row with different lowercase superscripts (a, b, c) are significantly different at *P* < 0.05

*CFU* colony forming unit

## Discussion

This study demonstrated significant improvements in metabolic health, antioxidant capacity, immune function, inflammatory status, and gut barrier integrity, suggesting that *Spirulina platensis* exerts physiological health benefits in working dogs. Of particular relevance, the most pronounced health benefits were consistently observed in the high-inclusion level group (SP2), indicating that the benefits are proportional to the inclusion level.

### Nutritional status parameters and fecal characteristics

In our study, no significant changes were observed in body weight, daily food intake, body condition score (BCS), fecal score, and fecal moisture among experimental groups. However, temporal changes over the study were noted for daily food intake and fecal moisture indicating natural physiological variation rather than dietary treatment as in previously reported studies [54, 55]**.** The lack of significant differences among dietary treatments or treatment × time interaction suggests that *Spirulina platensis* under the current experimental conditions did not markedly influence nutritional status parameters or fecal characteristics. These findings may be attributed to the use of adult dogs (2–3 years) that already reached their mature body size. Interestingly, the absence of significant alterations in these parameters suggests that *Spirulina* supplementation even with higher inclusion level (0.08 g/kg bodyweight/day) is well tolerated and did not cause any short-term adverse effects on appetite, or gastrointestinal function. This finding is very important for the practical application in diets of dogs. Similar findings are in agreement with previous reports which noted no significant alterations on nutritional status parameters or fecal quality following *Spirulina* supplementation in adult dogs [7, 21]. Despite this, *Spirulina plantensis* supplementation had notable positive effects on several physiological, immunological, gut health parameters, suggesting that its main role extends beyond nutritional status parameters and fecal characteristics in dogs.

### Serum biochemical indices

Regarding serum biochemical indices, *Spirulina* supplementation significantly influenced serum protein profile, including total protein, albumin, and globulin, particularly in the higher inclusion level of the *Spirulina* group (SP2) and mainly at the end of the experimental period. Notably, dogs receiving the higher inclusion level of *Spirulina plantensis* powder recorded the most pronounced increase, particularly at the end of the study (W7), compared to the other groups. These findings are consistent with the well-documented previous reports describing *Spirulina’s* bioactive components, such as phycocyanin, vitamins (especially B-complex), minerals, and linoleic acid, and its superior protein quality and quantity as a potent immunostimulant and modulator of hepatic protein synthetic capacity [24, 56, 57].

In a similar manner, lipid metabolism responded to the *Spirulina* supplementation in the diet. A dose-dependent effect was noticed, with the higher inclusion level (SP2) group showing the most pronounced outcomes. Notably, the lower inclusion level (SP1) group showed a beneficial effect, ranking second in terms of lipid profile modulation. This was reflected by a significant reduction in TC, TAG, LDL and VLDL levels, accompanied by a marked increase in HDL concentration at both the middle (W3) and end of the experiment (W7). These results are in line with the previous studies, which similarly demonstrated *Spirulina’s* hypolipidemic activity and its potential role in lipid metabolism [58–61]. In particular, Stefanutti et al. [11] demonstrated significant reductions in triglyceride concentrations in dogs following *Spirulina* supplementation at inclusion level ranging between 0.06 and 0.08 g/kg body weight/day. According to the literature, the hypolipidemic effects of *Spirulina* could be explained by many biological processes. C-phycocyanin, the principal protein constituent of *Spirulina*, has been proposed to enhance the activity of glutathione peroxidase (GSH-Px) and superoxide dismutase (SOD), thereby scavenging free radicals and inhibiting lipid peroxidation. In addition, C-phycocyanin has been reported to suppress the formation of nicotinamide adenine dinucleotide phosphate (NADPH) and NADH, as well as inhibit NADPH oxidase expression, mechanisms that may collectively underlie the hypolipidemic effects of *Spirulina* [61, 62]. Another bioactive compound isolated from *Spirulina*, the glycolipid H-b2, has been shown to reduce pancreatic lipase activity, consequently decreasing fat digestion and absorption within the intestinal lumen [61, 63]. Moreover, gamma-linolenic acid (GLA) derived from *Spirulina* was reported to regulate the cholesterol metabolism through its role in esterification processes [64]. Given that *Spirulina* represents a low-fat, low-calorie protein source, its consumption may attenuate hepatic triglyceride (TG) synthesis while enhancing hepatic triglyceride lipase and lipoprotein lipase activities, thereby improving the lipid profile [64, 65]. Furthermore, algal supplementation has been associated with a reduction in intestinal cholesterol absorption and bile acid reabsorption within the ileum [66, 67].

*Spirulina* also exerted a positive effect on hepatic and renal functions. Serum ALT and AST activities were significantly reduced in *Spirulina*-supplemented groups (SP1 and SP2), indicating the modulation of the liver status and reduced hepatocellular stress. The best results were observed in the higher inclusion level group (SP2). However, the lower inclusion level group (SP1) also showed a considerable improvement, occasionally comparable to the high dose group. These outcomes support the hepatoprotective role of *Spirulina* reported in previous studies [68, 69]. The improvement in liver enzyme activities observed following *Spirulina* supplementation may be attributed to its potent antioxidant properties, which protect hepatic tissues from oxidative damage [70, 71].

Regarding renal parameters, *Spirulina* supplementation led to a significant reduction in serum urea levels, reflecting enhanced renal performance. This support the nephroprotective role of *Spirulina* reported by previous studies [72–74]. The observed modulatory effects for kidney function tests are most likely attributable to the reinforcement of the endogenous antioxidant defense mechanisms and the attenuation of lipid peroxidation processes [75]. Although serum creatinine levels did not differ statistically between groups, it remained within normal physiological limits, supporting the absence of any renal adverse impacts. This pattern may reflect improved nitrogen handling and renal excretory efficiency without affecting glomerular filtration as measured by creatinine [76]. Urea is sensitive to short-term alterations in protein metabolism and renal clearance and may therefore respond earlier to dietary and physiological conditions [77, 78].

Beyond its metabolic effects, *Spirulina* supplementation markedly improved the antioxidant activity and lipid peroxidation of treated dogs, reflecting a potentiated antioxidant defense system. The significant increase in total antioxidant capacity (T-AOC) together with the reduction in malonaldehyde (MDA) level, suggests a strong free radical scavenging activity associated with *Spirulina* supplementation. These findings align with the previous research highlighting the antioxidant potential of *Spirulina,* which has been attributed to its rich composition of phycocyanin, Beta-carotene and other bioactive compounds [11, 59, 79, 80]. Similarly, Stefanutti et al. [11] reported that *Spirulina* supplementation for dogs at 0.06–0.08 g/kg body weight/day significantly enhanced antioxidant activity. C-phycocyanin was reported to confer protection against oxidative stress through scavenging of reactive oxygen species (ROS) and suppression of lipid peroxidation in liver microsomes. In addition, the carotenoid fraction of SP (yellow-to-red pigments) can enhance antioxidant activity by decreasing oxygen-induced lipid peroxidation and preventing intracellular ROS accumulation [81, 82].

While a number of serum biochemical parameters showed significant differences between treatment groups, all values were within established reference ranges for healthy dogs. Such changes do not indicate sick alterations but rather suggest subclinical or adaptive metabolic responses that are frequently seen in canine nutritional and supplement interventional studies. Implemented dietary supplementation with microbe-derived antioxidants induced beneficial biochemical changes in plasma parameters [83] without any negative clinical effects in healthy Beagle dogs. Likewise, vitamin D3 supplementation modified serum 25-hydroxy vitamin D and other hematological variables in healthy dogs with biochemistry still within normal ranges [84]. These trends support the interpretation of statistically significant biochemical shifts as modulation of metabolic processes rather than clinically adverse events. However, the biological and clinical significance of such changes requires careful consideration in interpretation and long-term studies.

### Gut health-related nutrigenomic response

Gut health is a dynamic and multifactorial process governed by the coordinated interaction between intestinal structure, resident microbiota, and dietary components. Together, these elements maintain mucosal integrity, support immune function, and ensure efficient nutrient utilization [85–87].

Circulating RNA represents extracellular transcripts released from tissues and immune cells into the bloodstream and has been increasingly used as a minimally invasive approach to assess nutrigenomic and inflammatory responses [88].FABP2 is a well-established circulating biomarker of intestinal epithelial integrity [89, 90], while occludin-related transcripts in serum are considered indirect indicators of tight-junction regulation and gut barrier modulation [89]. The use of serum-based circulating RNA was selected to enable repeated, non-invasive assessment of gut health-related nutrigenomic responses in working dogs, where intestinal tissue sampling is ethically and practically limited [88]. Moreover, this approach is supported by evidence demonstrating that noninvasive circulating biomarkers can reliably reflect intestinal barrier integrity and gut functional status, as shown by Linsalata et al. [91], who successfully identified gut barrier–related disease subtypes without reliance on intestinal tissue sampling as I-FABP, IL-6, IL-8, and TLR-4 using serological tests. In addition, Górecka et al. [92], who identified tight junction proteins such as occludin as reliable biomarkers of intestinal barrier leakage, underscoring the validity of using circulating tight junction–related molecular markers to assess gut integrity noninvasively. Consistent with our study, Chen et al. [90] validated circulating FABP2 and IL-6 mRNA as reliable noninvasive biomarkers reflecting intestinal epithelial injury and barrier dysfunction in both clinical (30 human neonate sample) and experimental models (in SPF BALB/c mice).

In this respect, there was a significant upregulation of occludin mRNA levels in *Spirulina*-supplemented groups, particularly at the higher inclusion level. Similar finding has been reported with *Spirulina* supplementation in rodent and *invitro* models [93, 94]. In addition, serum fatty acid binding protein-2 (FABP2) showed significant downregulation following *Spirulina* supplementation. FABP2 is an intracellular protein expressed in enterocytes; its presence in circulation is generally associated with enterocyte injury or increased intestinal permeability [95]. Therefore, the recorded reduction of FABP2 in *Spirulina*-supplemented groups (SP1 and SP2) may reflect improved gut barrier integrity and reduced epithelial leakage. This interpretation agrees with earlier studies describing serum FABP2 as a sensitive biomarker of intestinal injury [96, 97]. Our study provides the first evidence, to our knowledge, of reduced serum FABP2 levels following *Spirulina* supplementation in working dogs. This suggests an improvement in gut barrier integrity, which appears to contradict the tissue-specific up-regulation of FABP2 reported by Abdelfatah et al. [12]. This discrepancy may highlight the critical difference between FABP2 expression in damaged enterocytes and its subsequent leakage into circulation.

In terms of inflammatory and immune biomarkers, Interleukin-6 (IL-6) expression is significantly down-regulated in *Spirulina-*supplemented groups, reflecting a marked anti-inflammatory effect. IL-6 is a key pro-inflammatory cytokine that mediates the acute phase response and immune activation. *Spirulina'*s bioactive compounds, such as phycocyanin, may be responsible for the anti-inflammatory effects which are linked to mechanisms such as inhibiting the Nuclear Factor-κB (NF-κB) pathway, which is a key regulator of inflammation [20]. This agrees with the previous reports where *Spirulina* supplementation reduced IL-6 expression and systemic inflammation [20, 93, 94]. Similarly, Tumor necrosis factor receptor (TNFR)-associated factor 6 (TRAF6) expression was significantly reduced following the administration of *Spirulina.* TRAF6 is a central adaptive protein involved in toll-like receptor and interleukin-1 receptor (IL-1R) signaling leading to nuclear factor-kappa B (NF-κB) activation, which is vital for immune cell development, activation, and tolerance. However, NF-κB activation or downstream signaling events were not directly assessed in the present study; therefore, pathway-level inhibition cannot be confirmed. Moreover,TRAF6 is critical for the proper development and function of B cells, T cells, and macrophages.TRAF6 is an adaptor protein involved in Toll-like receptor and interleukin-1 receptor signaling cascades that are functionally linked to NF-κB activation and immune regulation [98]. To our knowledge, there are no previous studies directly reporting the effect of *Spirulina plantensis* on TRAF6 expression. However, similar downregulation of TRAF6 has been recorded with other nutraceuticals [99, 100]. While reduced TRAF6 expression may be consistent with altered inflammatory signaling dynamics, the absence of direct pathway analysis precludes definitive conclusions regarding NF-κB modulation. Accordingly, the present findings should be interpreted as evidence of treatment-associated changes in inflammatory signaling markers rather than confirmation of specific intracellular pathway inhibition.

### Fecal sIgA and calprotectin

Calprotectin and sIgA have been suggested to be the non-invasive markers of canine intestinal health [101]. sIgA represents the primary immunoglobulin synthesized by gut-associated lymphoid tissue (GALT), and it plays a crucial role in protecting against pathogenic infections, blocking antigen entry into the epithelium, and regulating the selection and maintenance of commensal bacteria [21]. In the current study, the higher inclusion level group of *Spirulina* (SP2) markedly increased fecal sIgA levels at the end of the experiment compared with the lower inclusion level or control groups, indicating enhanced GALT activity. Prior investigations have shown that dietary *Spirulina* or its extracts stimulate an increase in intestinal IgA levels [21, 102, 103]. This immunostimulatory effect of *Spirulina* may be attributed to its bioactive compounds like phycocyanin and polysaccharides. These compounds activate GALT and stimulate Toll-like receptors (TLR-2 and TLR-4) which enhance B cell differentiation into IgA-secreting plasma cells [21, 104]. This significant increase in fecal sIgA observed in the *Spirulina* supplemented groups particularly the higher inclusion level group (SP2) at the end of the experiment can suggest a dose and time-dependent immunostimulatory effect. This is consistent with the threshold concept described by World Health Organization [105]. According to WHO, there is a threshold level below which there is no measurable response. This “no-observed-effect level” (NOEL) indicates the minimum exposure needed to trigger a detectable physiological or biochemical change. Therefore, when the lower inclusion level of *Spirulina* did not produce a significant change in the fecal sIgA level, this may indicate that this inclusion level did not exceed the threshold required for biological stimulation.

Regarding fecal biomarkers of gastrointestinal inflammation, Calprotectin accounts for about 60% of the protein content within neutrophil cytosol. Inflammatory damage to the mucosal barrier permits neutrophil leakage, resulting in the release of calprotectin into the gut lumen and its subsequent fecal excretion [106]. The results of the present study revealed a significant reduction in calprotectin levels in *Spirulina-*supplemented groups reflecting an improvement in intestinal inflammatory status and epithelial integrity*.* Although direct reports measuring fecal calprotectin after *Spirulina plantensis* supplementation are scarce, the significant reduction observed in the present study appears biologically plausible. This is consistent with the known anti-inflammatory mechanisms of *Spirulina,* which include suppression of pro-inflammatory cytokines(IL-6,TNF-α) and inhibition of NF- NF-ĸB [63, 107]. Accordingly, these findings collectively suggest a decrease in neutrophil-driven intestinal inflammation and improved mucosal integrity indicated by decreased fecal calprotectin, a well-established biomarker of intestinal inflammation [108]**.**

### Fecal microbiota analysis

A noticeable improvement was observed in intestinal microbial count in groups supplemented with *Spirulina plantensis,* particularly the higher inclusion level group. This was evidenced by the increased *Lactobacillus* count and reduced Coliform count. These findings are in line with previous research that stated that *Spirulina* could improve the gut microbiota stability by increasing *Lactobacillus* spp. and decreasing *E. coli* populations[12, 23, 109, 110]. *Spirulina* naturally produces soluble polysaccharides that exert a prebiotic-like effect, bypass digestion, and fermented by gut bacteria [21, 23]. Moreover, tocopherols and C-phycocyanin present in Spirulina possess antimicrobial properties, acting against several pathogenic bacterial species, including *Klebsiella pneumoniae, Pseudomonas* spp., *E. coli*, and *Enterobacter* spp. *Salmonella Typhi*, and *Proteus vulgaris*[23, 111]. The positive impact of *Spirulina* on gut immune function may, in turn, affect the composition and activity of the gut microbiota, and a bidirectional relationship is also plausible.

It's worth mentioning that throughout the study, the group with a higher inclusion level of *Spirulina* (SP2) maintained the best performance in most of the measured parameters, followed by the lower inclusion level group (SP1), reflecting a clear dose-dependent response.

It is important to note that outcomes showing no statistically significant differences should not be interpreted as evidence of the absence of a biological effect. Given the relatively small sample size, the study may have been underpowered to detect modest but biologically meaningful changes. Therefore, non-significant findings should be considered inconclusive rather than indicative of no treatment effect, and future studies with larger cohorts are warranted to clarify these observations.

While this study provides robust evidence for the beneficial effects of *Spirulina platensis*, several limitations must be considered. First, the study was conducted on a homogeneous population of working dogs with a relatively small number, which may limit the direct generalizability of the results to other canine populations, such as sedentary, geriatric, or juvenile dogs, or those of different breeds. Hence, further studies are needed on larger populations to confirm and extend the results. Second, the experimental study duration, while sufficient to detect significant physiological changes, does not provide data on the long-term sustainability of these effects or potential impacts on chronic disease incidence. Third, although the study was conducted in working dogs, performance evaluation was limited to body weight and body condition score. Objective functional performance and workload-related indicators (e.g., endurance capacity, activity monitoring, or task-specific performance metrics) were not assessed. The clinical evaluations, including body condition scoring (BCS) and fecal scoring, were performed by a single veterinarian who was aware of the treatment groups, which may introduce observer bias. Nevertheless, the use of a single trained evaluator applying standardized scoring systems may reduce interobserver variability. Recent evidence indicates that interobserver agreement for BCS and muscle condition scoring in dogs can be variable, whereas within-observer consistency is generally higher when standardized criteria are applied by experienced personnel [112]. Moreover, established BCS systems have been widely validated and shown to be reliable when consistently used [113]. Despite these mitigating factors, the lack of blinding remains a limitation, and future studies should incorporate blinded assessments and/or multiple evaluators to further enhance objectivity. Fourth, the analysis, though comprehensive, was largely focused on specific biochemical and molecular biomarkers. Employing multi-omics analysis (e.g., metabolomics, metagenomics) could elucidate novel mechanistic pathways influenced by *Spirulina.* Fifth, the use of serum-derived circulating RNA to assess gut health-related gene expression rather than direct intestinal tissue analysis. Therefore, the nutrigenomic findings should be interpreted as indirect indicators of gut barrier modulation. Sixth, the gut microbiota assessment was performed using culture-based methods, which provide targeted information on selected cultivable bacterial groups but do not capture the full microbial diversity or functional potential of the intestinal ecosystem. Therefore, microbiota-related conclusions should be interpreted cautiously, and future sequencing-based studies are recommended. Finally, the precise bioavailability and metabolism of *Spirulina's* bioactive compounds in the canine model remain to be fully characterized.

## Conclusion

In the present study, short-term dietary supplementation with *Spirulina platensis* did not result in statistically significant alterations in nutritional status parameters, including body weight, daily food intake, and BCS or fecal characteristics, including fecal score and fecal moisture. The absence of statistical significance in these parameters should not be interpreted as the absence of a biological effect, particularly given the limited sample size and study duration. Nonetheless, significant modulations were observed in several analyzed biochemical, antioxidant, inflammatory and molecular markers after supplementation with spirulina relative to control groups (all values within the physiological reference ranges). Interestingly, these findings represent functional metabolic and immunological adaptations rather than pathological changes. Dose-dependent trends were particularly evident, where a higher inclusion level typically displayed greater responses in all measured endpoints throughout the 7-week trial. Serum-based molecular findings, including modulation of OCLN, FABP2, and inflammatory signaling markers, were interpreted as systemic indicators associated with gut integrity and inflammatory balance rather than direct measures of intestinal transcriptional activity. Similarly, observed changes in selected culturable fecal bacterial populations reflect targeted microbial shifts rather than comprehensive microbiota remodeling. Overall, the data suggests that *Spirulina platensis* supplementation may contribute to systemic metabolic, antioxidant, and immunomodulatory regulation in working dogs. However, given the exploratory nature of the study and the relatively small sample size, these findings should be considered preliminary. Larger, longer-term, and tissue-validated investigations are warranted to confirm these effects and determine their clinical and performance-related relevance.

## Data Availability

All data generated or analyzed during this study are included in this published article.

## Abbreviations

BCS: Body condition score
TC: Total cholesterol
TAG: Triacylglycerol
HDL: High-density lipoprotein
LDL: Low-density lipoprotein
VLDL: Very low-density lipoprotein.
AST: Aspartate aminotransferase
ALT: Alanine aminotransferase
T-AOC: Total antioxidant capacity
MDA: Malondialdehyde
CFU: Colony-forming unit.
OCLN: Occludin
FABP2: Fatty acid binding protein-2.
IL-6: Interleukin -6.
TRAF6: TNF receptor- associated factor 6
